# Preclinical biodistribution and toxicology assessment of an AAV5-based subretinal modifier gene therapy for retinitis pigmentosa

**DOI:** 10.3389/fmed.2025.1679619

**Published:** 2025-10-29

**Authors:** Arun Upadhyay, Selina Drag, Pushpendra Singh, Kalpana Rajanala, Rasappa Arumugham

**Affiliations:** Ocugen Inc., Malvern, PA, United States

**Keywords:** retinitis pigmentosa, modifier gene therapy, nuclear hormone receptor, hNR2E3, subretinal delivery

## Abstract

Retinitis pigmentosa (RP) is a multifactorial disease caused by mutations in over 100 genes, affecting ~1 in 4,000 people worldwide, and is characterized by abnormalities in the rod and cone photoreceptors. Gene therapy approaches are promising and have been established to provide an unprecedented treatment option for genetic diseases caused by mutation(s) in a single gene. However, traditional gene therapy approaches are not pragmatic for RP due to its heterogeneous genetic background. To this end, modifier gene therapy is a unique approach in which a transgene can correct or rescue the detrimental effects caused by mutations in unrelated gene(s). Nuclear receptor subfamily 2 group E member 3 (NR2E3) is a nuclear hormone receptor that exhibits the characteristics of a modifier gene. In preclinical studies, subretinal delivery of NR2E3 rescued the RP phenotype by resetting the molecular pathways to restore normal ocular structure and function in multiple models of RP disease. For clinical studies, AAV5*-hNR2E3* was designed for the subretinal delivery of the *hNR2E3* gene through the AAV5 vector in RP patients. In this study, we evaluated the safety and biodistribution (delivery and expression) of a gene therapy candidate, OCU400 (AAV5*-hNR2E3*), in Göttingen minipigs delivered via the subretinal route. Administration of the product at all dose levels was well tolerated, resulting in ocular changes that were minor and mostly related to the dosing procedure, with no significant signs of systemic or ocular toxicity. AAV5*-hNR2E3* vector was preferentially localized to the target retinal tissues with minimal to no exposure to systemic organs and tissues after subretinal administration. This localized delivery efficiently transduced retinal cells, where the delivered transgene produced the NR2E3 protein.

## Introduction

Retinitis pigmentosa (RP) is a debilitating ocular disease that leads to progressive retinal degeneration affecting 1/4,000 people worldwide (1). Patients affected by RP develop night blindness and loss of peripheral vision due to the loss of rod and cone photoreceptors in the initial stages. Further disease progression leads to degeneration of cone photoreceptors and eventually results in complete loss of vision (2, 3). RP is a complex multifactorial disease with >3,000 mutations in over 100 genes (4, 5) with genetic causes that can be autosomal dominant (32 genes, 5–25% RP cases), autosomal recessive (68 genes, 5–20% RP cases), or X-linked (6 genes, 5–15% RP cases) (4, 6). The latest research has helped decipher the genetic architecture and global distribution of RP (7, 8).

With the advancement of gene therapy approaches, it is possible to rescue and even reverse disease progression in inherited genetic disorders, thereby allowing a normal, healthy life for the affected individuals (9, 10). Recently, approaches such as optogenetics, which aim to reengineer retinal cells by introducing light-sensitive proteins into functioning neurons, are under development. A multi-characteristic engineered opsin (MCO-010) (11), administered via intravitreal injection, has been shown to restore visual behavior in mouse models (12) and improve visual function in advanced RP patients (13). The gene replacement therapy approach, in which a dysfunctional gene is replaced with a functional copy, has the potential to treat monogenic cases with recessive mutations (14). However, since more than 100 genes are implicated in RP, each gene would require its own unique gene therapy. Moreover, disease phenotypes of RP vary greatly by age of onset, severity, and rate of progression. These variations can result from allelic heterogeneity, environmental influences, genetic modifiers, or a combination of all of these factors. In addition, the genetic basis is unknown in approximately 40% of cases (15). Therefore, traditional gene therapy is not a pragmatic approach. In modifier gene therapy, a transgene capable of rescuing the detrimental effects due to dysfunctional, unrelated gene(s) is a promising solution to these problems, independent of a genetic defect (16–18). The modifier gene therapy restores cellular homeostasis in the disease state by resetting the altered cellular networks with an overall positive output (16–20).

**Figure 1:**
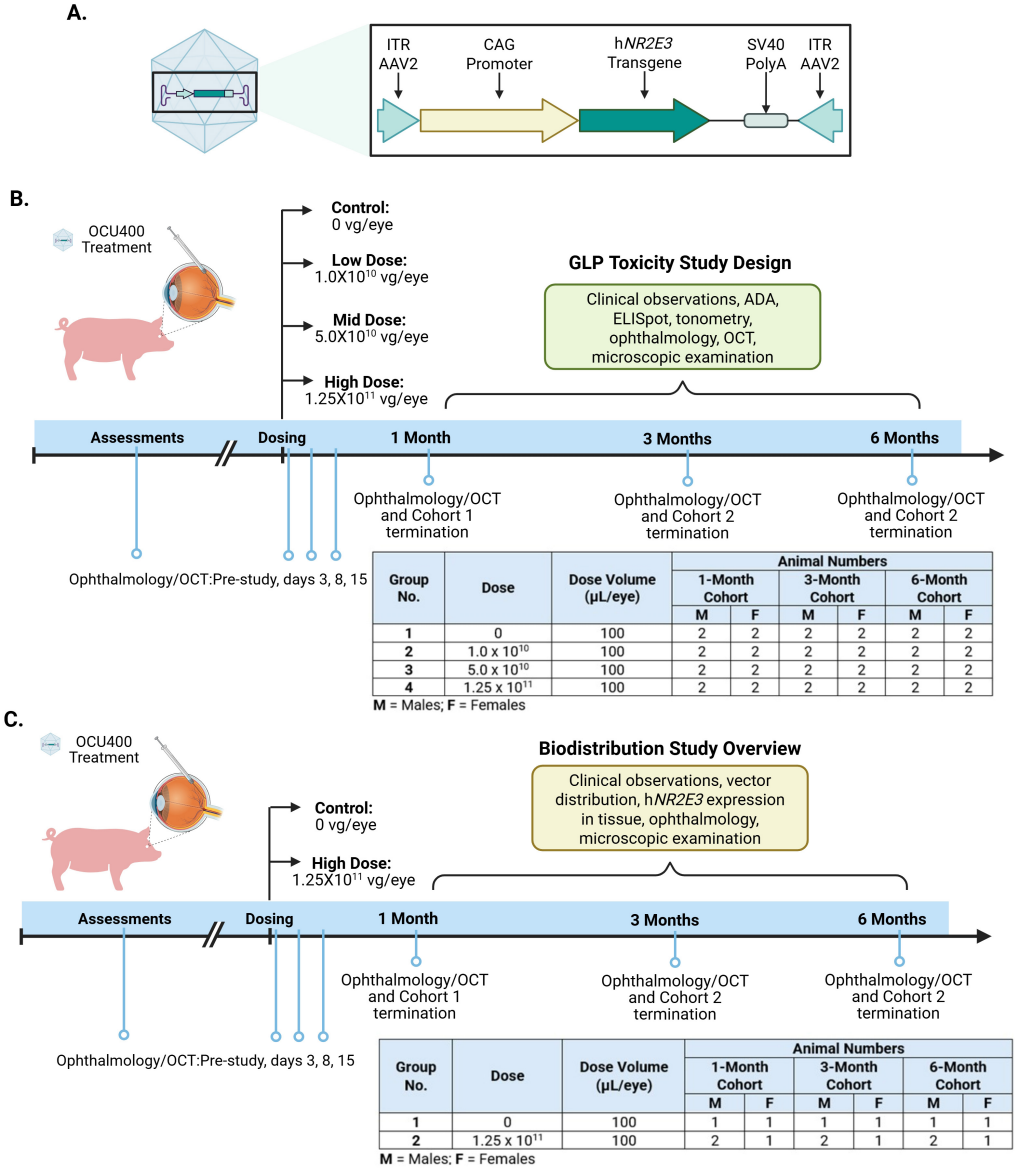
OCU400 Transgene, Toxicology and Biodistribution Study design. **(A)** The transgene elements are labeled in the boxed area: ITR2 sequence, CAG promoter, NR2E3 transgene, PolyA SV40 sequence, and ITR2 sequence. Schematic depicting the design of **(B)** GLP toxicology study **(C)** Biodistribution study. Image was generated using BioRender.

Nuclear hormone receptors such as nuclear receptor subfamily 2 group E member 3 (NR2E3) exhibit the aforementioned modifier gene characteristics (20–29). NR2E3 has a dual role of activator and repressor, and its activity is essential for the proper development and function of rod and cone photoreceptor cells (30–32). Specifically, NR2E3 regulates cone cell proliferation in retinal progenitors and promotes rod-cell differentiation in postmitotic rod photoreceptors by suppressing cone genes and activating rod-specific genes (32–34). More than two decades of research in multiple mouse models of RP with different underlying mutations (rd1, Rho^−/−^, Rho^P23H^, rd16, and rd7) has established that NR2E3 can rescue retinal degeneration and RP disease progression (19, 26, 35, 36). In preclinical studies, various doses of AAV5-hNR2E3 administered during the early to intermediate stages of retinal degeneration in rd7 or RhoP23H^+/−^ mice models restored homeostasis by resetting key Nr2e3-associated pathways, and resulted in improved photoreceptor survival and function (35, 36). Based on the success of these studies, the modifier gene therapy AAV5*-hNR2E3* was developed to treat RP disease in humans via a single subretinal injection (Figure 1). The AAV5 serotype selectively delivers the hNR2E3 transgene to the retinal cells in the human eye (14, 37).

As retinal cells are post-mitotic, the gene therapy product is likely to be sustained, and potential adverse effects might be long-lasting. Therefore, in preparation for a phase I/II clinical trial, the (Good Laboratory Practice (GLP)-toxicology and biodistribution studies are critical to establishing the safety profile of the therapy in an appropriate model. To evaluate the delivery and expression of AAV5*-hNR2E3*, we performed a biodistribution study in Göttingen minipigs, which have a similar anatomy to the human eye (38–40). Moreover, the NR2E3 protein has high homology (>90%) between these species (NCBI database). AAV5*-hNR2E3* was administered subretinally, and the biodistribution of the vector was evaluated in ocular and non-ocular tissues. We also conducted a toxicology study using the same animal model to establish the safe dose range and to investigate the correlation between clinical findings and dose strength.

## Materials and methods

### Animals

Göttingen minipigs (*Sus scrofa*) were obtained by Charles River Laboratories. At the initiation of dosing, the animals were 3–5 months of age with a target weight of 7–15 kg and were acclimated to the facility prior to dosing. Post-acclimatization, animals were randomly assigned to groups and divided into three cohorts (1, 3, or 6 months) for each dose level. The Biodistribution study was a non-GLP study wherein the animals were randomly assigned to either a control group or a treatment group and divided into three cohorts (1-, 3-, or 6-month follow-up).

The toxicology study was planned in accordance with GLP regulations. It was reviewed and approved by the Charles River Laboratory Institutional Animal Care and Use Committee (IACUC) prior to execution. Up to three animals of the same sex and dosing group were housed in stainless steel cages with vinyl-coated mesh floors. Animals were provided with enrichment and fresh fruits and vegetables daily in a temperature—and humidity-controlled room with a 12-h light/dark cycle. They were fed a controlled diet and had access to water *ad libitum*, except during designated procedures.

### Assessment of safety and biodistribution of subretinally administered AAV5*-hNR2E3* in minipig: study design

For ophthalmic gene delivery, an adeno-associated virus serotype 5-based vector containing the human NR2E3 (AAV5*-hNR2E3*) gene expression cassette was designed, as the AAV5 capsid has been shown to have specificity for retinal cells. The sequence of the DNA corresponding to the hNR2E3 transgene corresponds to the known human *NR2E3* sequence (NCBI: NM_014249.3) (Figure 1A). AAV-hNR2E3 produced in HEK293 cells was used in this study.

To determine the potential ocular toxicity and immunogenicity of the gene therapy product, 100 μL of vehicle or AAV5*-hNR2E3* at doses 1.0 × 10^10^, 5.0 × 10^10^, and 1.25 × 10^11^ vg/eye were administered via a single subretinal injection in Göttingen minipigs. The animals were evaluated at 1-, 3-, and 6-month post-dosing for toxicity and adverse health effects (Figure 1B).

Assessing the biodistribution of gene therapy vectors is crucial, as off-target delivery would have a long-lasting impact that could lead to undesirable chronic adverse effects on patient health. Therefore, we evaluated the distribution, persistence, and clearance of vector following a single 100 μL subretinal injection of AAV5*-hNR2E3* at a dose of 1.25 × 10^11^ vg/eye, the highest dose used in a toxicology study. Ocular and non-ocular tissues were collected at 1-, 3-, and 6-month post-dosing to determine the tissue distribution of the vector (NR2E3). Target and non-target ocular tissues, systemic organs, and blood samples were analyzed using quantitative polymerase chain reaction (qPCR) and enzyme-linked immunosorbent assay (ELISA) methods. In addition, General health was monitored throughout the study, and potential ocular toxicity was monitored by ophthalmic examinations (fundoscopic and biomicroscopic) (Figure 1C).

### Reagents

Dosing procedure-related materials included prednisone, Kenalog^®^-40, 0.5-cc syringe, 29-gauge half inch needle, tobramycin, ketamine, dexmedetomidine, butorphanol, isoflurane/sevoflurane, povidone-iodine, Zephiran™ (benzalkonium chloride), mydriatic drops, proparacaine, MedOne MicroDose™ Injection kit 1 mL with adapter, a MedOne PolyTip^®^ cannula 25 g/38 g [25 g × 28 mm cannula with 38 g × 5 mm tip], and an Alcon^®^ Viscous Fluid Control Pak attached to the Alcon^®^ Constellation Vision System, buprenorphine, and atipamezole.

The quantitative polymerase chain reaction (qPCR) primers and probe mixes were custom-ordered from Integrated DNA Technologies (IDT). Other reagents included TaqMan Gene Expression Reaction Mix (Applied Biosystems, 4369016, 4369510, and/or 4369542), TE buffer (Invitrogen, 12090015), and DMSO (ThermoFisher, D12345). Background matrix DNA (minipig genomic DNA) and study-specific tissues were procured at Charles River Laboratories. DNA was isolated from study tissues using the Maxwell^®^ RSC Instrument and Maxwell^®^ RSC Blood DNA Kit (Promega, AS1400) and quantified by spectrophotometer (SpectraMax). Samples were analyzed using the QuantStudio 7 Flex Real-Time PCR system.

Meso Scale Discovery (MSD)-based enzyme-linked immunosorbent assay (ELISA) reagents for transgene protein expression included MSD Sulfo-Tag NHS Ester (Meso Scale Discovery, R91AO-1) and EZ-Link™ Sulfo-NHS-LC-Biotin, No-Weigh™ Format (Thermo Scientific, A39257) for antibody labeling; protease inhibitor cocktail (Sigma-Aldrich, P8340), cOmplete ULTRA protease inhibitor cocktail (Roche, 05892970001), PhosSTOP phosphatase inhibitor cocktail (Roche, 04906847001), cell lysis buffer (Cell Signaling Technologies, 9803S), and the Pierce BCA protein assay kit (Thermo Scientific, 23225 or 23227) for homogenization of tissues and protein determination; and 96-well SMALL-SPOT^®^ streptavidin plate (MSD, L45SA-X), human NR2E3 recombinant protein (custom), MSD Diluent 100 (MSD, R50AA), MSD Diluent 30 (MSD, R50AB-2), MSD Blocker A Kit (MSD, R93AA), MSD Read Buffer T with surfactant (MSD, R92TC), Tween-20 (Sigma, P-1379), and Phosphate Buffered Saline (PBS; Gibco, 70013 and 20012). ADA ELISAs used AAV5*-hNR2E3* as the coating material, anti-AAV5 intact particles (ADK5a, mouse monoclonal, IgG2a/K; Progen) for positive controls, and pooled normal minipig serum (BioIVT) for negative control, horseradish peroxidase (HRP), and tetramethylbenzidine (TMB).

For ELISpot analysis, sodium heparin tubes and gentle MACS C tubes were used for sample collection and isolation. Other reagents included FBS, 10% DMSO, AIV-V medium, RPMI-1640 medium, and the Porcine interferon gamma (IFN-γ) ELISpot Kit (R&D Systems).

Electroretinography (ERG) materials included ketamine, dexmedetomidine, butorphanol, mydriatic drops, and Zephiran™ (benzalkonium chloride). For blood collection, K_2_ potassium ethylenediaminetetraacetic acid (K_2_EDTA) tubes and sterile DNase-free and RNase-free polypropylene tubes were used. Animals were euthanized using ketamine, glycopyrrolate, acepromazine, and sodium pentobarbital.

### Anesthesia

Following a suitable fasting period, a cocktail of ketamine (10 mg/kg), dexmedetomidine (0.04 mg/kg), and butorphanol (0.2 mg/kg) was administered via intramuscular injection. During the procedures, animals were intubated and maintained on oxygen. Reversal agent Atipamezole 0.4 mg/kg was administered when considered necessary.

### Dosing and subretinal injections

An oral corticosteroid regimen was administered to all animals prior to and post-subretinal dosing to minimize procedure-related inflammation. Animals received 1 mg/kg of corticosteroid prednisone for 3 days prior to dosing, then in a tapering dose once daily for 4 weeks (1 mg/kg for the first week, 0.75 mg/kg for the second week, 0.5 mg/kg for the third week, and 0.25 mg/kg for the fourth week). After a fasting period, animals were given an intramuscular sedative cocktail consisting of 5 mg/kg ketamine, 0.04 mg/kg dexmedetomidine, and 0.22 mg/kg butorphanol, followed by a mix of isoflurane/oxygen or sevoflurane/oxygen by endotracheal tube. The periorbital region was cleaned with povidone-iodine and sterile water, and the conjunctivae were flushed using diluted benzalkonium chloride (Zephiran™). Mydriatic drops and 0.5% proparacaine were applied, with additional drops applied as needed.

The AAV5*-hNR2E3* test article was thawed on wet ice, formulated to the desired strength, and kept on ice for the duration of the procedure. A 100 μL of vehicle or AAV5*-hNR2E3* was administered with a 1 mL MicroDose™ injector (MedOne) and a 25g/38g PolyTip^®^ cannula (MedOne) using the Alcon^®^ Constellation Vision System pump (Alcon). Dose formulations were administered as transvitreal, single-bleb injections in ~30 s in the superior region or the region supra-nasal to the optic nerve. Both eyes were examined by slit-lamp biomicroscopy and/or indirect ophthalmoscopy to evaluate any abnormalities. In addition, animals received bilateral subconjunctival injections of 4 mg Kenalog^®^-40 (triamcinolone acetonide, 40 mg/mL) in a dose volume of 100 μL using a 0.5-cc syringe with an attached 29-gauge half inch needle. All injections were followed by the application of topical tobramycin twice daily on the day before and day after the dose was administered. Prior to dosing and as appropriate following the procedure, 0.01 mg/kg buprenorphine was administered intramuscularly. Reversal agent atipamezole (0.4 mg/kg) was administered at the conclusion of all procedures unless animals were deemed sufficiently awake.

### Animal health assessment

General health assessments, including mortality and moribundity checks, food consumption evaluations, detailed clinical observations, and individual body weight measurements, were closely monitored. In the event of the development of ocular inflammation during the study, animals were administered topical mydriatic agents (1% atropine), topical steroid drops (1% prednisolone acetate), subconjunctival steroid (triamcinolone acetonide 40 mg/mL), systemic analgesic (buprenorphine), or systemic corticosteroid.

### Blood collection

Blood was collected by venipuncture on the day of necropsy for vector biodistribution and transgene protein expression analyses. For vector biodistribution, whole blood was collected into K_2_EDTA tubes, inverted several times, and then immediately transferred into sterile DNase-free and RNase-free polypropylene tubes and frozen on dry ice for analysis by qPCR. For transgene expression, whole blood was collected using the same anticoagulant, processed to plasma, and then frozen on dry ice for analysis by ELISA.

### Vector biodistribution analysis

Vector biodistribution in various minipig tissues and biofluids was analyzed by qPCR using primers and probes specific to human *NR2E3*. The DNA extraction was performed on the collected specimens using the Maxwell DNA Extraction Kit (Promega), and the extraction efficiency was over 70%. Calibration standards and quality control samples were prepared with 35 to 1 × 10^7^ copies/reaction using linearized hNR2E3 plasmid in a background of 0.14 μg/μL minipig gDNA. No template controls (NTC) and a control at the limit of detection (5 copies/reaction) were analyzed in each run. Study samples were diluted to 0.04 μg/μL (blood and retina) or 0.14 μg/μL (all other matrices) unless the amount of DNA was too low to normalize by concentration, in which case the samples were not diluted. All study samples were tested for interference by spiking 200 copies/reaction of reference DNA into study samples.

### Transgene protein expression analysis

The amount of human NR2E3 protein present in different tissues was determined using an MSD-based ELISA. Mouse anti*-*hNR2E3 antibodies were labeled with ruthenium and biotin. Minipig tissues were isolated and homogenized in a cell lysis buffer containing protease and phosphatase inhibitor cocktails, followed by protein estimation.

### Ophthalmic examinations

Ophthalmic examinations, including indirect ophthalmoscopy (fundoscopic) and slit lamp (biomicroscopic) evaluations, were performed at regular intervals. Intraocular pressure was measured using a TonoVet^®^ rebound tonometer.

### Electroretinography

Electroretinography (ERG) was performed once prior to dosing and during weeks 4, 13, and 26 post-dosing. After fasting, animals were anesthetized using a cocktail of 10 mg/kg ketamine, 0.04 mg/kg dexmedetomidine, and 0.2 mg/kg butorphanol. Animals were intubated and maintained on oxygen during the ERG procedures. Animals were adapted to the dark for a minimum of 30 min. Mydriatic drops were administered to each eye, and peripheral stay sutures were employed to maintain eye position during the recording, following a flush of the conjunctivae with diluted Zephiran™ (benzalkonium chloride) and administration of the topical anesthetic. Each ERG occasion included both scotopic (dark-adapted) and photopic (light-adapted) phases. Baselines were corrected as needed to correct waveform drift and analyzed for a-wave and b-wave amplitudes, implicit times, and latency.

### Clinical pathology

Clinical pathology samples were collected by venipuncture prior to dosing and at regular time points post-dosing. Hematology samples were collected as whole blood using EDTA anticoagulant tubes. Coagulation samples were collected into sodium citrate anticoagulant tubes and processed to plasma. Clinical chemistry samples were collected after overnight fasting into serum separator tubes without anticoagulant to collect serum.

### Anti-drug antibody (ADA) analysis

Serum and aqueous humor samples were collected and analyzed by ELISA for the presence of anti-AAV5 and anti-hNR2E3 antibodies at different time points. Blood samples were collected by venipuncture in serum separator tubes without anticoagulant and processed to serum. Aqueous humor samples were collected from both eyes 6 ± 1 days prior to scheduled euthanasia. To collect aqueous humor, animals were fasted and then administered an intramuscular injection of 0.03 mg/kg dexmedetomidine as a pre-anesthetic followed by anesthetization with an isoflurane/oxygen mixture as needed. The collection was performed using a 1 mL syringe with a 30-gauge needle. Eyes received a mydriatic agent, then conjunctivae were flushed with Zephiran™ (benzalkonium chloride). Topical anesthetic drops (0.5% proparacaine) were administered, and at the conclusion of the procedure, tobramycin topical antibiotic ointment was applied, and 0.3 mg/kg of the reversal agent atipamezole was administered by intramuscular injection unless the animal was sufficiently awake. A target volume of 50 μL per eye was collected and stored at −80 °C until analysis.

ADA analysis was performed using a tiered approach. A screening assay was performed first to identify initial positive or negative samples. Then a titration assay was performed to estimate the titer of ADA for the positive samples. In the ADA ELISA, minipig anti-AAV5 and anti*-*hNR2E3 antibodies present in minipig serum and aqueous humor samples were bound to the immobilized antigen. Detection of the bound antibodies was performed with goat anti-swine immunoglobulin G (IgG) horseradish peroxidase (HRP) conjugated secondary antibody, followed by the addition of metabolized tetramethylbenzidine (TMB) to the microtiter plate. The resulting chromophore was read at 450 nm (A_450nm_) by a spectrophotometer, and the absorbance correlated with the level of antibodies present in the samples.

### ELISpot analysis

Enzyme-linked immunospot (ELISpot) analysis was performed on peripheral blood mononuclear cells (PBMCs), lymphocytes, and spleen cells. Cells and tissues were collected once prior to dosing and on days 29, 92, and 183. Whole blood was collected via venipuncture into tubes with the anticoagulant sodium heparin and processed to PBMCs using a Ficoll-Paque PLUS gradient density method. They were then analyzed for the detection of interferon gamma (IFN-γ) using an analytical ELISpot for T-cell responses to peptide pools of AAV5 vector capsid and hNR2E3 protein. Results were reported as IFN-γ spot-forming cells (SFCs) per million PBMCs.

Spleen and mandibular lymph nodes were collected at the time of euthanasia using cell culture clean procedures. Samples were collected into gentle MACS C tubes containing 8 mL of RPMI-1641 medium and processed into a single-cell suspension for detection of IFN-γ by the ELISpot method, as performed for PBMCs. Spleen and mandibular lymph node cells were stimulated *in vitro* using 200,000 cells/well and eight different peptide pool conditions: negative control of AIM-V medium, positive control of 5 μg/mL phytohemagglutinin (PHA) from *Phaseolus vulgaris*, and 2 and 5 μg/mL of 4 different AAV5 peptide pools and 2 different hNR2E3 peptide pools. Results were reported as IFN-γ SFCs per million cells.

### Terminal procedures

Post 1, 3, and 6 months of dosing, animals were euthanized by the following procedure: 1. intramuscular injection of a ketamine (22 mg/kg), glycopyrrolate (0.01 mg/kg), and acepromazine (1.1 mg/kg) cocktail, 2. intravenous injection of sodium pentobarbital, and 3. exsanguination by incision of the axillary or femoral arteries. Animal tissues were embedded in paraffin, sectioned, mounted on glass slides, and stained with hematoxylin and eosin for histological analysis and microscopic evaluation by a board-certified veterinary pathologist.

### Data analysis

The total number of animals used in the study was the minimum required to properly characterize the effects of the gene therapy product. Data were collected, and descriptive statistics, including arithmetic means and standard error of the mean (SEM), were determined as appropriate. Statistical analyses were performed on data using appropriate tests such as the unpaired *t*-test or two-way analysis of variance (ANOVA).

## Results

### AAV5*-hNR2E3* subretinal administration was safe and well-tolerated with no effect on the general health of the animals

To determine the potential ocular toxicity and immunogenicity of AAV5*-hNR2E3*, different doses of the product were administered via a single subretinal injection in Göttingen minipigs. The animals were evaluated at 1-, 3-, and 6-month post-dosing for toxicity and adverse health effects (Figure 1B). General health was monitored throughout the study, and potential toxicity was evaluated using tonometry, ERG, clinical pathology, ophthalmic examinations [fundoscopic, biomicroscopic, and optical coherence tomography (OCT)], anti-drug antibody analysis (by ELISA), immunogenicity (by enzyme-linked immunosorbent spot or ELISpot), histology, and microscopic evaluations. Clinical chemistry parameters, including liver enzymes [alanine transaminase (ALT), aspartate aminotransferase (AST), alkaline phosphatase (ALP), and gamma-glutamyl transferase (GGT)], bilirubin levels, kidney function markers (creatinine and urea nitrogen), electrolytes (sodium, potassium, and chloride), and other metabolites such as glucose, cholesterol, and triglyceride levels, were also evaluated in the animals. In addition, hematology parameters to assess blood components such as red and white blood cells, hemoglobin, hematocrit, and platelets, with additional counts for specific white cell types like neutrophils and lymphocytes, and coagulation tests to evaluate blood clotting function through measurements like activated partial thromboplastin time, prothrombin time, and fibrinogen levels were also assessed. All these assessed parameters remained within normal ranges before and after dosing. Isolated minor elevations in reticulocyte (RETIC) values were observed pre-study in a few animals and were considered either a result of the large volume of blood collected for pre-study PBMC samples for ELISpot analyses (20 mL) or were incidental. In addition, no obvious changes were observed in any hematology parameters following corticosteroid treatment. The minor differences in hematology parameters were considered not related to AAV5*-hNR2E3*- based on their small magnitude, inconsistent direction, absence of a dose response, general overlap of individual values with the range of control and/or baseline values, and/or were of a magnitude of change commonly observed in minipigs under similar study conditions. AAV5*-hNR2E3*-related changes in any coagulation or clinical chemistry parameters were not observed during the study. Elevated creatine kinase (CK) values were seen in many animals during the pre-study and were considered related to the animals struggling during blood collection procedures. Increases in aspartate aminotransferase (AST) were noted pre-study for some animals. Given the isolated nature of the increases and the lack of a dose response, this was considered unrelated to AAV5*-hNR2E3* administration. In this study, two animal deaths occurred during data collection at the final 6-month time point (1 in the control and 1 in the high-dose group). Upon investigation, it was determined that deaths occurred due to the procedure for in-life measurements and were not related to the AAV5*-hNR2E3* treatment. In one control animal, breathing difficulties occurred immediately following extubation (removal of the endotracheal tube) during recovery from anesthesia after week-26 spectral domain optical coherence tomography (SD-OCT) imaging, so the animal was humanely euthanized on day 179. Similarly, one animal from the high-dose group was found dead on day 181 following clinical signs of decreased activity, abnormal breathing sounds (including labored breathing, wheezing, and pinned back ears when the animal was placed in a sling), reduced appetite, weakness, and pale skin that were noted on days 180 and 181. Both deaths were considered accidental and due to laryngeal perforation at the time of intubation during preparation for week-26 SD-OCT imaging. Both animals had a grossly visible perforation in the ventral larynx, accompanied by surrounding swelling, and fluid or material accumulation. Eyes from both animals were included in the evaluation of the 6-month cohort eyes.

**Figure 2:**
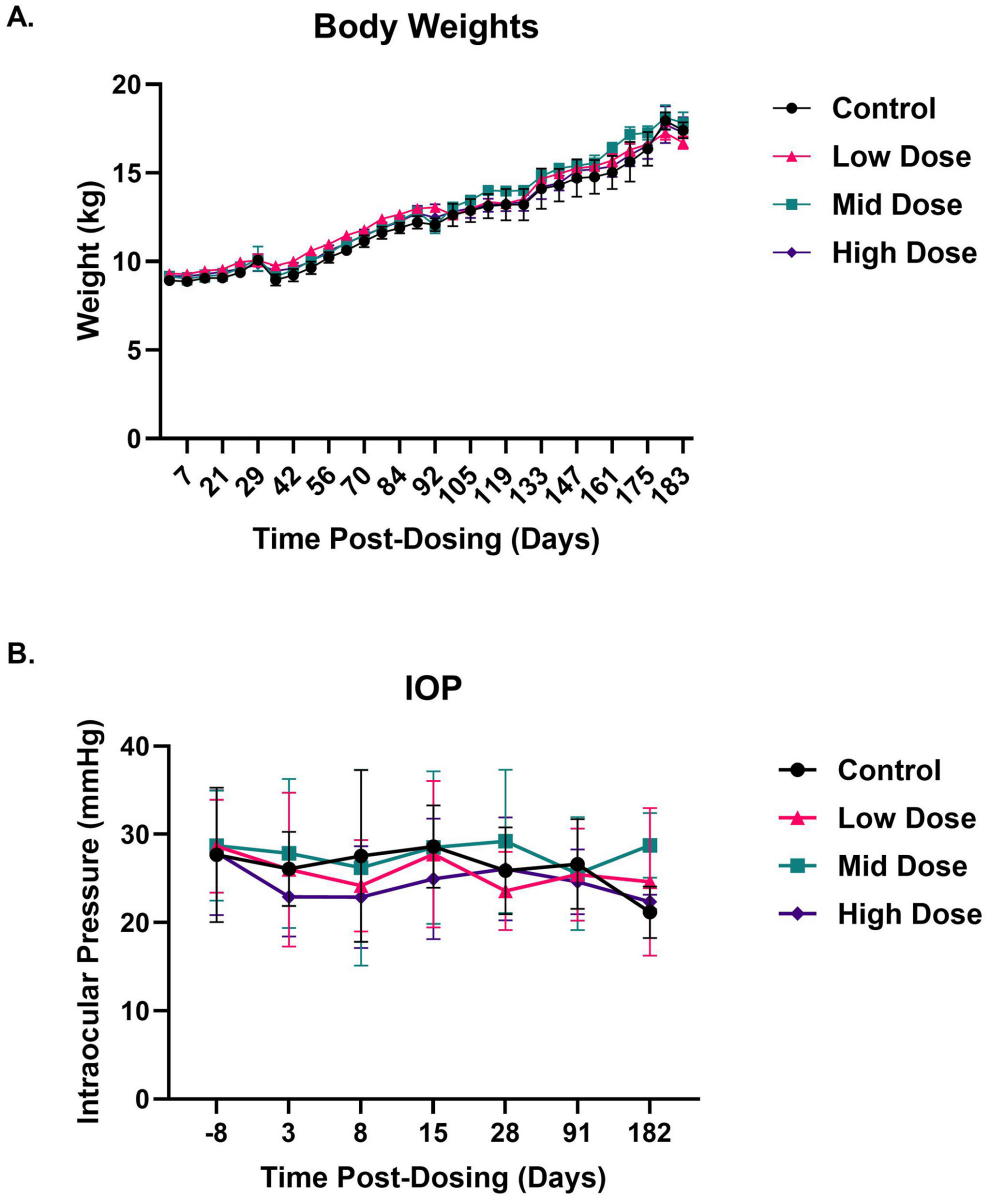
OCU400 administration did not affect animal health (body weight) in general or ocular health (intraocular pressure). As a part of the GLP toxicology study, the general health of the animals was evaluated. **(A)** Animal body weights showed a progressive increase over the study for all treatment groups, including those receiving different doses of OCU400, as well as the untreated control. Animals did not show any significant difference in weight gain among the sdifferent treatment groups. **(B)** Intraocular pressure (IOP) ranged from 20 to 35 mm Hg pre-dosing the animals, and the IOP values stayed within this range post-OCU400 administration during the study. IOP data showed higher fluctuation at early time points (days 3 and 8) post-dosing the animals. No statistically significant difference was observed. Black: control; Magenta: low dose; Cyan: mid dose; Indigo: high dose. *N* = 12 animals per dose group at 1 month, *N* = 8 animals per dose group at 3 months, and *N* ≥ 3 at 6 months post-dosing (total 48 animals at the beginning of the study). Error bars represent standard deviation.

AAV5*-hNR2E3* administration did not result in any changes in body weight or body weight gains as compared to control animals. Body weight remained relatively unchanged from the pre-dose in both control and treated animals, especially up to day 35. After day 35, body weight increased at a steady and comparable rate between the control and treated animals (Figure 2A). Transient fluctuations in intraocular pressure (IOP) (> ± 5 mm Hg) were frequently observed from occasion to occasion in eyes across all dose groups, including controls, as well as elevated IOP values (≥25 mm Hg). In the absence of any ocular inflammatory findings or ocular findings that could impact IOP, these changes were considered related to animal stress due to tonometry procedures (performed in conscious animals) and were not considered related to AAV5*-hNR2E3* administration (Figure 2B). Nonetheless, most of these findings were resolved within the first 2 weeks after dosing.

### Retina and vitreous humor exhibit the highest levels of AAV5*-hNR2E3* after subretinal delivery of the vector

**Figure 3:**
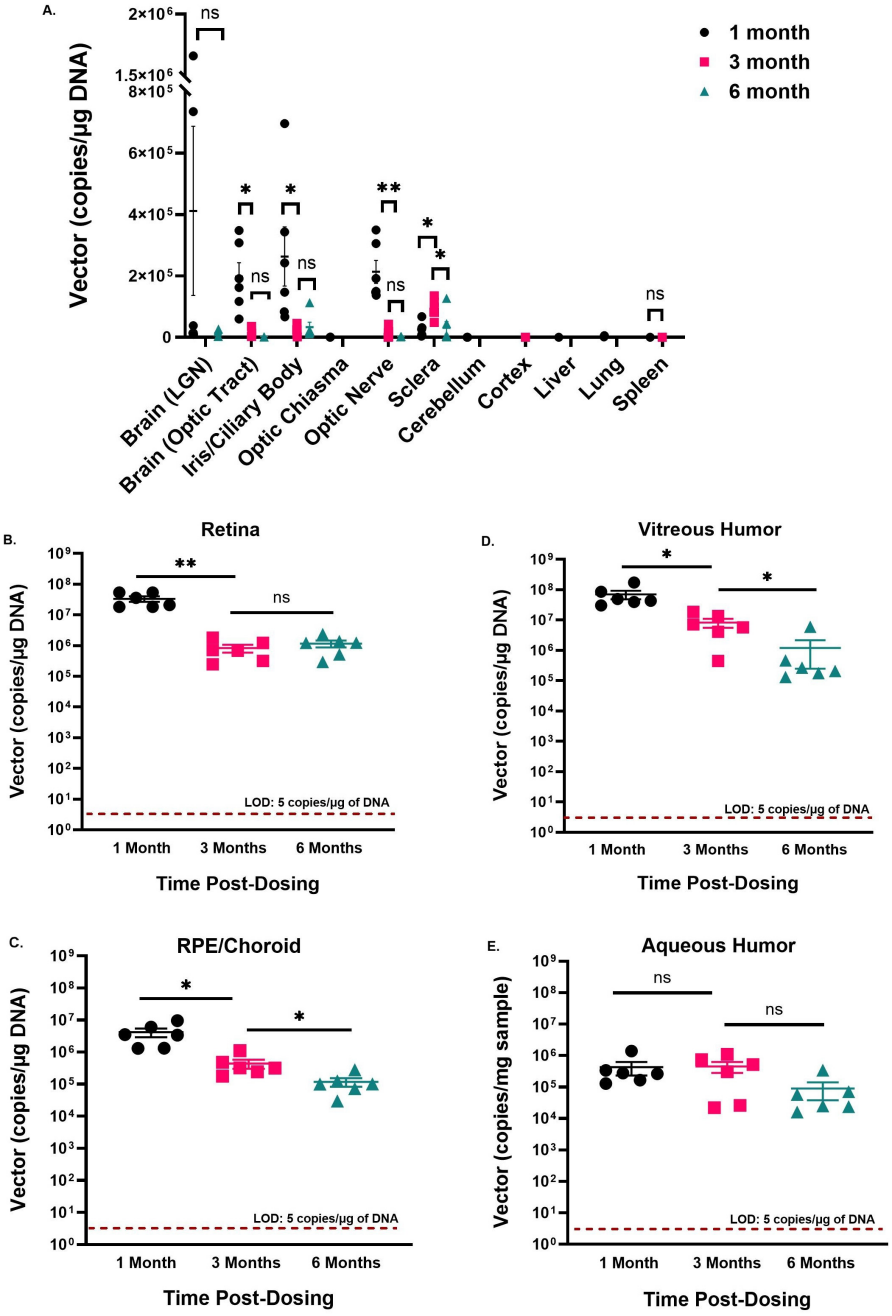
**(A–E)** Vector Biodistribution Analysis in Ocular and Non-Ocular Tissues. The vector biodistribution in the dosed animals administered a single dose of 1.25 × 10^11^ is represented. Each dot for the graph depicting non-ocular tissues represents one animal, and each dot represents one eye for the respective graphs depicting data from ocular tissues (Retina, vitreous humor, RPE/choroid, aqueous humor). The horizontal line represents the mean, and the error bars represent the standard error of the mean (SEM). Measurable quantities of vector were observed at only one time point in the optic chiasma, cerebellum, cortex, liver, and lung. ns = not significant; *p-value < 0.05; **p-value < 0.001; LGN, lateral geniculate nucleus. We did not include the control animals in this analysis as they are not expected to show any expression.

Vector biodistribution was quantified at 1-, 3-, and 6-month post-dosing by qPCR using specific primers and a probe against the transgene. The qPCR method was developed, and the validity of the primers and probe to selectively identify h*NR2E3* was established in different minipig matrices. No vector DNA was detected in any of the control animal matrices at any time point. Figure 3A shows the AAV5*-hNR2E3* vector distribution in different ocular and non-ocular tissues in treated animals. At 1 month, the vector was detected at low levels in the lung, spleen, and cerebellum, and these levels decreased to undetectable levels by 6 months. Moreover, low levels of AAV5*-hNR2E3* were observed in blood, lymph node, and liver at 1 month, which were cleared by 6 months. AAV5*-hNR2E3* was not detected in any reproductive tissue (i.e., ovary, testis, or uterus/cervix), heart, kidney, or thymus at any time.

Figures 3B–D show the distribution of AAV5*-hNR2E3* in retina, vitreous humor, and retinal pigment epithelium (RPE)/choroid, respectively. Retina and vitreous humor exhibited the highest levels of vector at 1 month, which decreased ~10-fold at 3 months. Vector levels in RPE/choroid were ~10-fold lower at 1 month compared to retina and vitreous humor. Interestingly, similar to retina and vitreous humor, RPE/choroid also exhibited a decrease in vector levels of ~10-fold at 3 months. Similar pharmacokinetics of the retina, vitreous humor, and RPE/choroid reflect the proximity of the ocular tissues. At 6 months, the level of vector was sustained in the retina; however, a further decrease was observed in the vitreous humor and RPE/choroid.

On the other hand, Figure 3E shows that the levels of AAV5*-hNR2E3* in the aqueous humor are drastically lower compared to those in the retina, vitreous humor, and RPE/choroid throughout the study. Owing to the low number of cells present in the aqueous humor, the vector copies detected could not be normalized to μg of DNA and were instead expressed as per mg of sample. AAV5*-hNR2E3* levels in aqueous humor remained unaltered from 1 to 3 months and exhibited a 5-fold decrease in 6 months.

### NR2E3 expression in the RPE/choroid progressively increased with time after dosing

All samples that were positive for the presence of vector were analyzed for NR2E3 protein expression. Many positive tissues (both ocular and non-ocular) showed NR2E3 expression below the lower limit of quantitation (LLOQ). In the RPE/choroid, NR2E3 protein was not detected at 1 month in any dosed animals. However, the protein showed a steady increase in the number of positive eyes from 0/6 to 2/6 and 6/6 at 1, 3, and 6 months, respectively.

**Figure 4:**
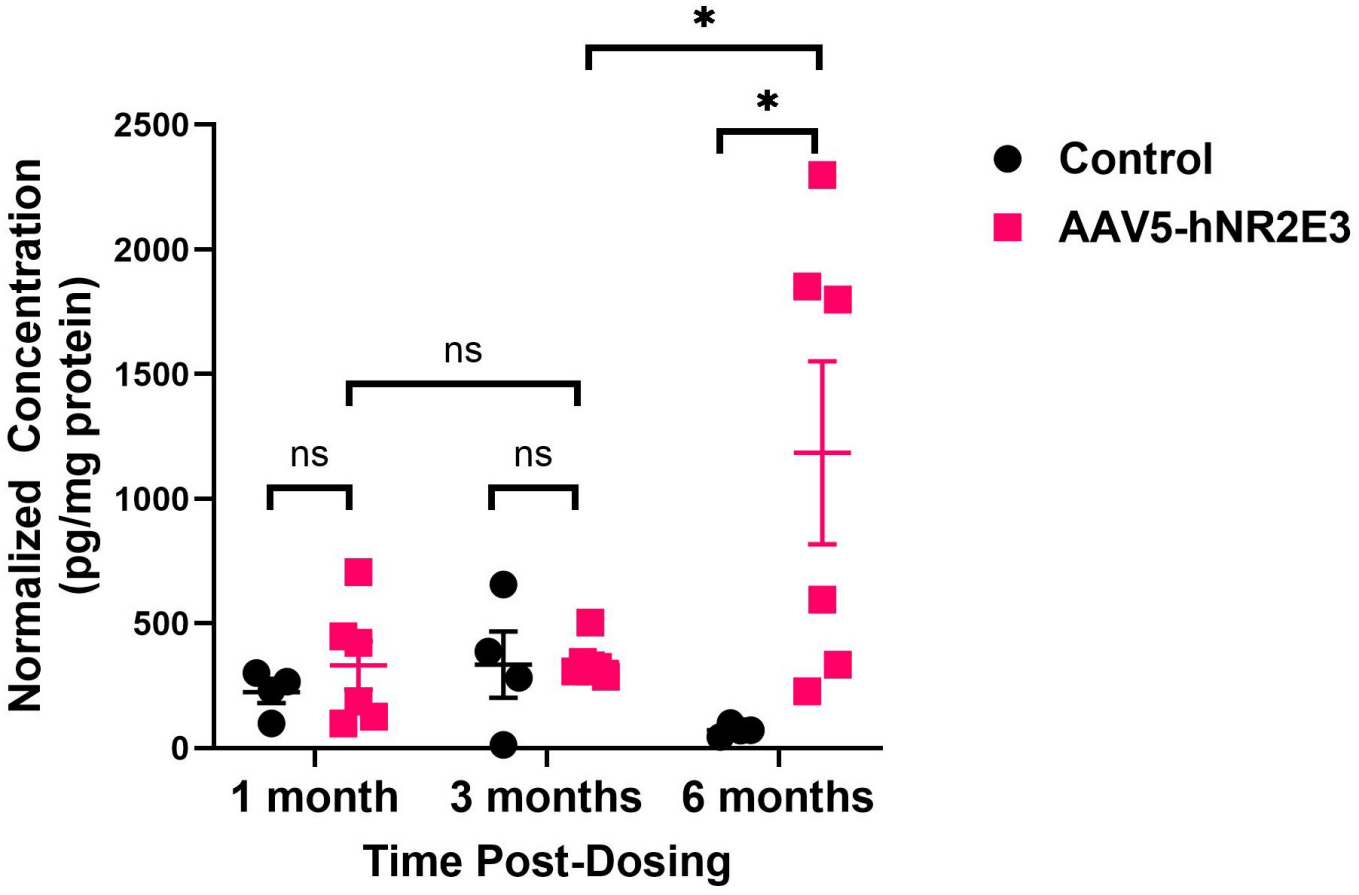
Transgene expression analysis. As part of a biodistribution study, the NR2E3 transgene protein expression in the retina was evaluated. Each dot represents data from one eye of the animal (Control group: four eyes of the two animals; Dose group: six eyes of the three animals). The horizontal line represents the mean, and the error bars represent the standard error of the mean (SEM). ns, not significant; *p-value < 0.05.

Retina tissue exhibited a significant amount of NR2E3 protein at 6 months. Figure 4 shows that NR2E3 protein expression in treated tissues is comparable to that of the controls at 1 and 3 months, but shows a dramatic increase at 6 months. The vector genomic DNA level showed a progressive decrease, whereas NR2E3 protein expression increased over time.

### AAV5*-hNR2E3* administration did not have an adverse effect on ocular function

Electroretinography (ERG) was used to measure ocular function in this study. In ERG, eyes are stimulated with light, and electric potential changes are recorded in the form of an electroretinogram. The a- and b-wave amplitudes and implicit times are deduced from the electroretinogram. Typically, the b-wave contains information on the electrical activity of bipolar cells, whereas the a-wave represents the response from cone and rod cells. These parameters indicate the rod and cone cell function and, therefore, the visual function of the eye.

**Figure 5:**
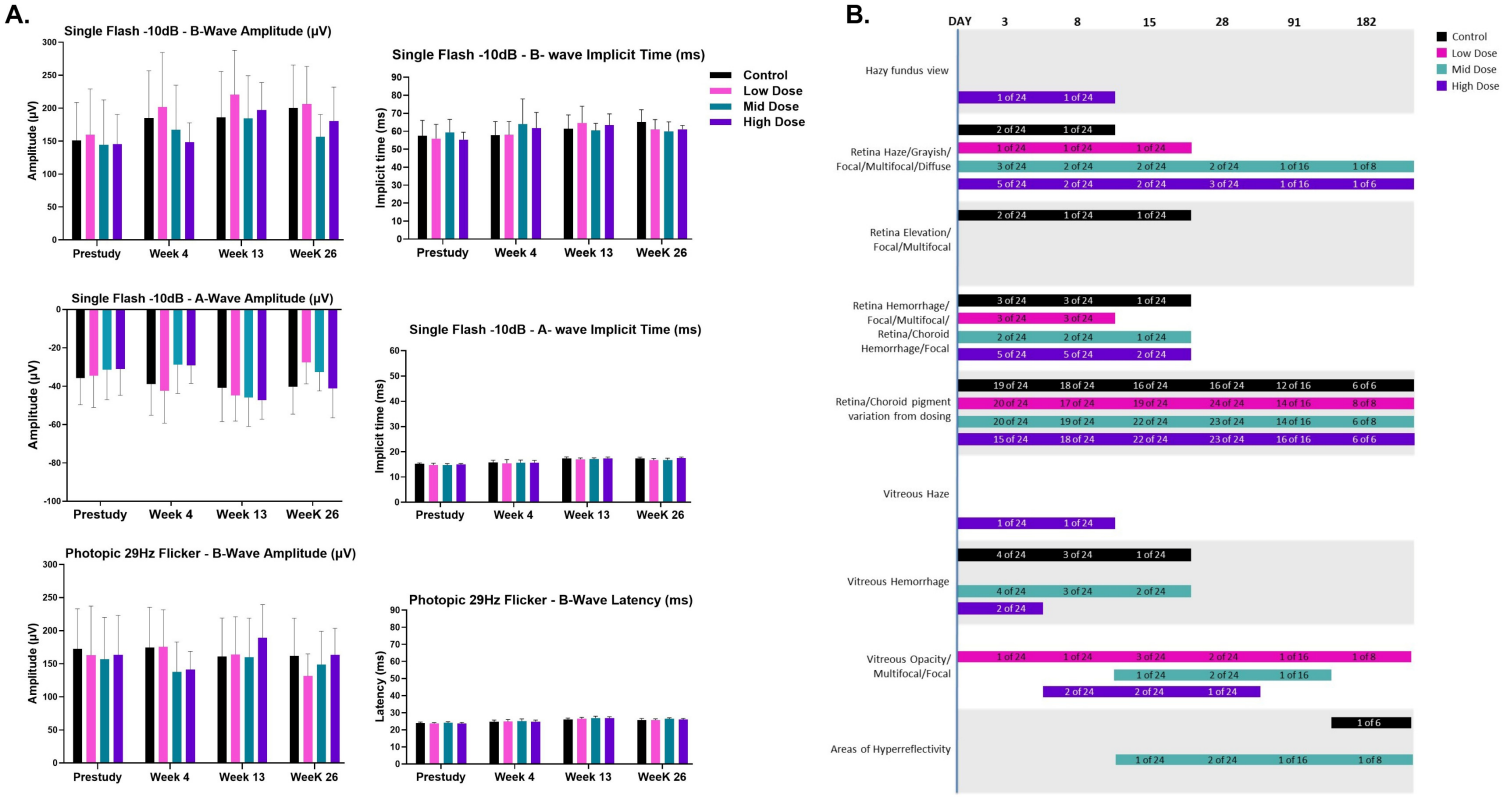
Evaluation of ERG and ophthalmic findings from GLP toxicology study. **(A)** Administration of OCU400 did not impact the Scotopic 10 dB B-wave, Scotopic 10 dB A-wave, and Photopic 29 Hz flicker B-wave ERG recordings compared to control animals. Black: control; Magenta: low dose; Cyan: mid dose; Indigo: high dose. *N* = 24 eyes per dose group at 1 month, *N* = 16 eyes per dose group at 3 months, and *N* = 8 eyes at 6 months post-dosing (total 48 animals at the beginning of the study). Error bars represent the standard deviation in the figure. **(B)** OCU400 resulted in dosing procedure-related ophthalmic findings. Ophthalmic examination revealed minor findings consistent with the subretinal dosing procedure. Most findings resolved within the first 2 weeks post-dosing, with recovery in the majority of remaining findings by the end of the study. Black: control; Magenta: low dose; Cyan: mid dose; Indigo: high dose.

Figure 5 shows the b-wave amplitude response ranges from ~145 to 220 μV and implicit time from ~55 to 65 ms under dark-adapted conditions when stimulated with a single flash of −10 dB. Under light-adapted conditions, the mean b-wave amplitude ranged from ~131 μV to 189 μV, and the implicit time ranged from 24 to 27 ms with a stimulus of 29 Hz flicker. These data show that bipolar cell activity remains unaffected between dark and light-adapted conditions. There were no AAV5*-hNR2E3* delivery-related changes in mean scotopic or photopic amplitudes and implicit times/latency observed during the study, as compared to values from pre-study and/or reference item-treated animals. Increases and/or decreases in ERG amplitudes were observed on occasion for some individual Scotopic or photopic tests for some animals; however, these were isolated events and were considered related to individual variability, and/or anesthesia levels. There is no significant difference in electrical communication in the eyes across different groups in dark and light-adapted conditions. Taken together, these data suggest that AAV5*-hNR2E3* administration is safe and does not adversely affect ocular function.

Ophthalmic examinations revealed that most eyes had chorioretinal pigmentary changes associated with the subretinal injection dosing procedure, where the subretinal blebs were created. This change (retina/choroid pigment variation from dosing) was secondary to the physical separation of the neurosensory retina from the retinal pigmented epithelium and is nearly always noted following bleb formation. Pigment variation naturally occurs when the neurosensory retina is elevated from the retinal pigmented epithelium, and this change can be exacerbated by *in situ* toxicity. In this study, some bleb areas were more prominent than others, which is a common finding following subretinal dosing. Regularly, a more prominent and small area of depigmentation can also be seen at the needle insertion site (where the needle enters the subretinal space). Up to day 28, the severity of the pigmentary changes was very slight to slight in many eyes, with moderate severity seen in 13/36 AAV5*-hNR2E3*-treated eyes. This finding was still present in the eyes of surviving animals on day 182, at a very slight to slight severity, with a generally similar severity to what was observed on day 91. Other findings related to the dosing procedure included transient retinal and/or choroidal hemorrhage, vitreal hemorrhage, conjunctival hyperemia/hemorrhage, and corneal opacities. Vitreal opacities were also observed in AAV5*-hNR2E3*-treated eyes, but were likely related to the dosing procedure. Approximately half of these cases also had vitreal hemorrhage, which often became whitish/grayish vitreal opacities over time. These hemorrhages were either caused by the minor iatrogenic trauma to the retina and/or choroid when blebs were created, or from the scleral ports at the level of the ciliary body where the instruments entered the posterior segment of the eye. None of the hemorrhages were noted after day 15, and one eye still had minor vitreal opacities on the last examination (day 182).

Multimodal spectral domain optical coherence tomography imaging (SD-OCT) findings revealed that most eyes had pigmentary changes in the bleb area (e.g., hyper-reflective dots, hyporeflective areas). A majority of eyes across dose groups had multifocal hyperreflective foci located in different retinal sublayers, generally in the outer retinal layers, including the outer plexiform layer (OPL) and the outer nuclear layer (ONL). Distortion of some retinal layers was seen in the bleb area in a majority of reference items and AAV5*-hNR2E3*-treated eyes, mainly in the photoreceptor (PR) layer and retinal pigment epithelium (RPE). Hyperreflective dots and/or accumulation of hyperreflective dots were observed in the vitreous of many eyes and sometimes correlated with ophthalmic findings of vitreal hemorrhage and/or vitreous opacities. Overall, animals in all treatment groups exhibited minor ophthalmic findings; a summary of ophthalmic observations for all the animals is presented in Figure 5B, which are commonly observed and known to be related to the subretinal dosing procedure (41, 42).

### AAV5*-hNR2E3* administration did not elicit a significant immune response

Anti-drug antibody (ADA) analysis is performed to evaluate the immune response against a drug product. Since AAV5*-hNR2E3* was administered subretinally, the local ADA response in the eye was evaluated in the aqueous humor, and the systemic ADA response was evaluated in the serum.

**Figure 6:**
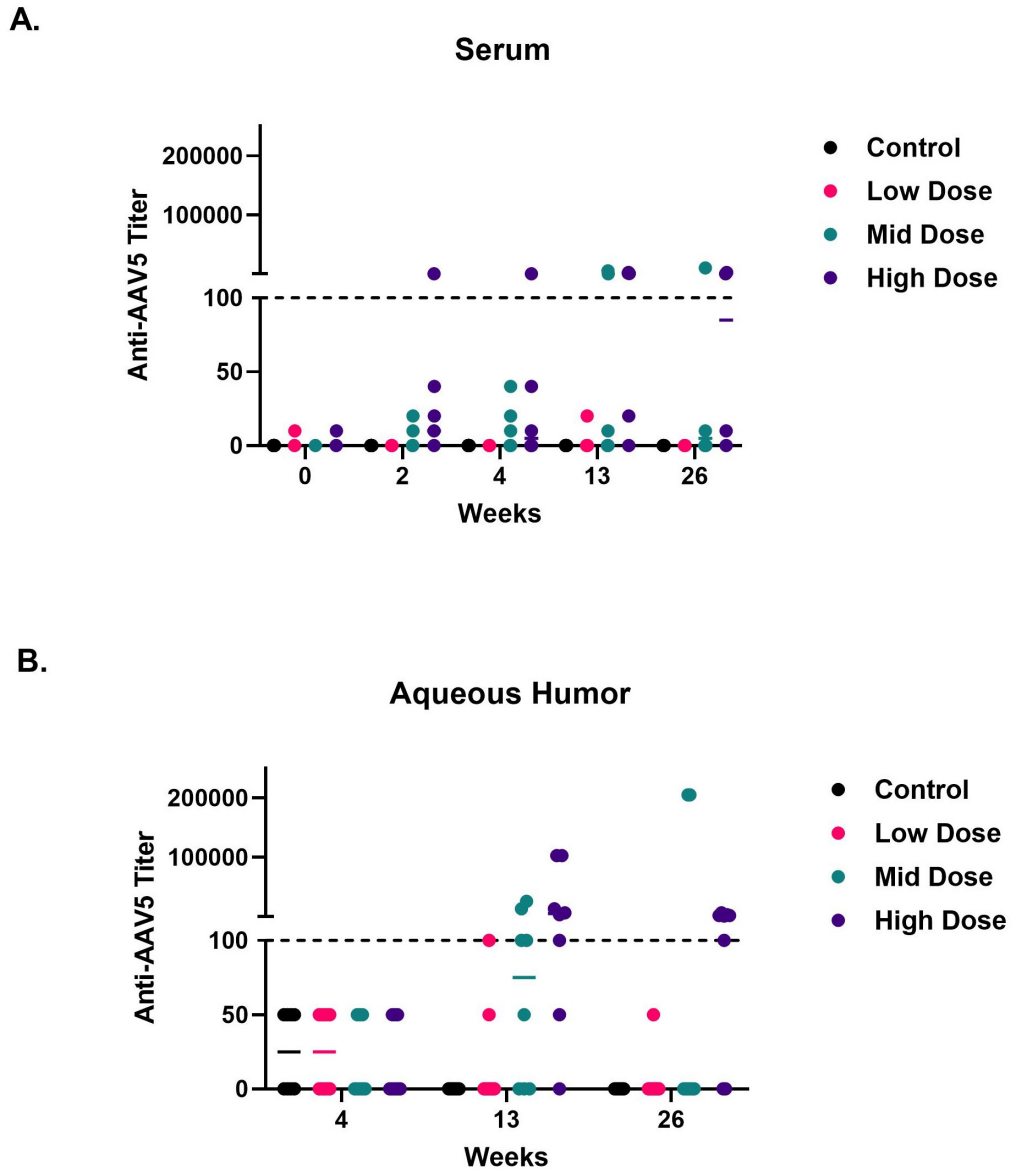
OCU400 resulted in anti-AAV5 ADA response in mid and high-dose groups. The results from the individual anti-AAV5 ADA response from the GLP toxicology study are presented. **(A)** Serum samples and **(B)** aqueous humor samples plotted with the median. The dotted line represents the threshold value of 100; values at or below this threshold were considered equivalent to background. Black: control; Magenta: low dose; Cyan: mid dose; Indigo: high dose. Each dot represents the data from one animal.

Figure 6A shows increased anti-AAV5 ADA titer in serum in a dose-dependent manner. Increased titers were observed in 25% of animals in the mid-dose group at 3 and 6 months and in ~8, ~17, ~63, and 50% of animals in the high-dose group at 2 weeks and 1, 3, and 6 months, respectively. Figure 6B demonstrates anti-AAV5 ADA titers in aqueous humor. Higher titers were present in 25% of eyes at the mid dose and ~63% of eyes at the high dose at 3 and 6 months. While AAV5*-hNR2E3* administration resulted in an anti-AAV5 ADA response, it did not show any corresponding systemic clinical signs or ophthalmic changes. Notably, no anti*-hNR2E3* ADA response was observed in either serum or aqueous humor of dosed animals.

**Figure 7:**
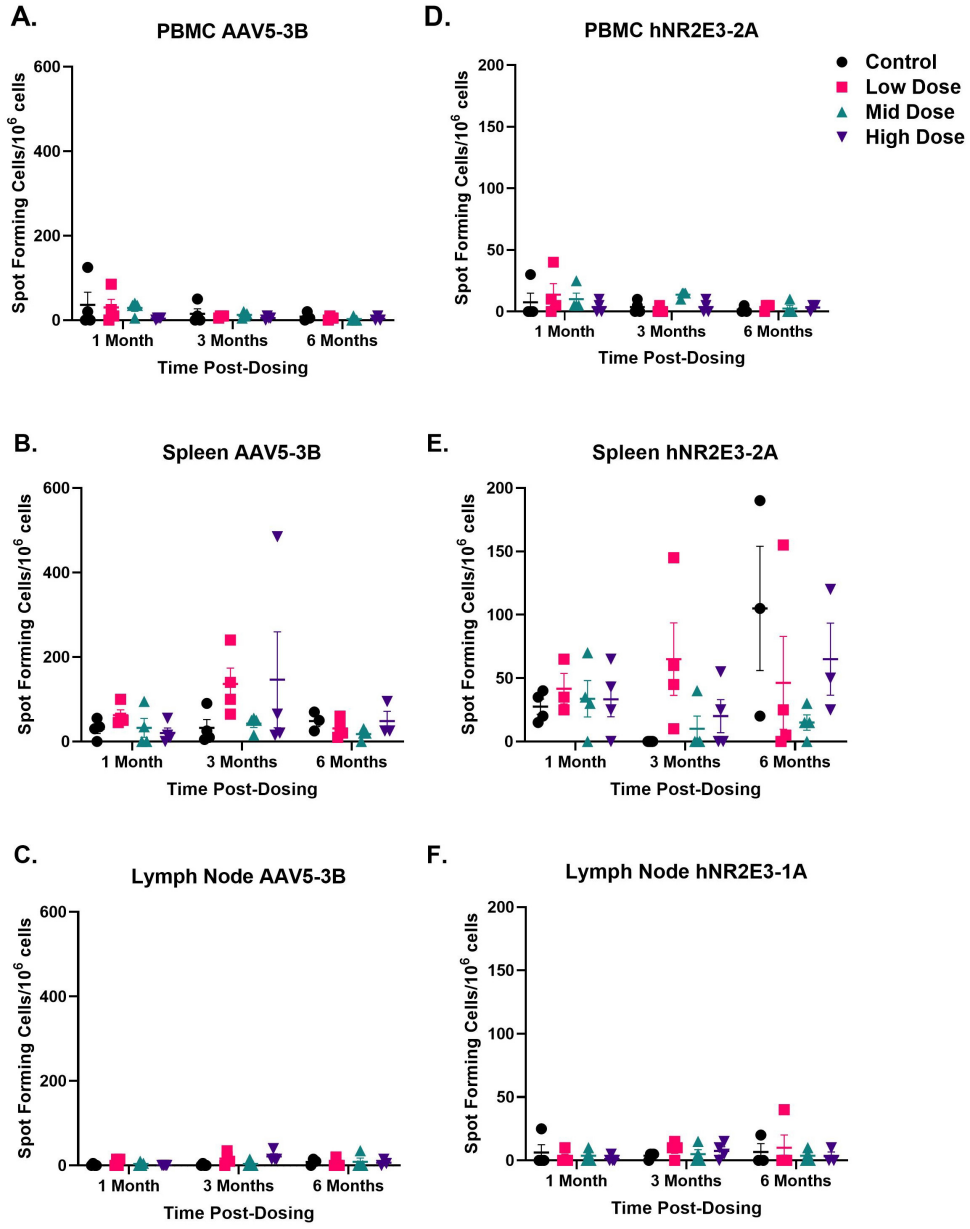
OCU400 did not elicit an immune response to AAV5 or hNR2E3 in PBMCs, splenocytes, or lymphocytes. **(A)** PBMCs did not show any immune response to AAV5. **(B)** Splenocytes appear to show an immune response compared to other cell types, but the result was within assay variability. **(C)** Lymphocytes did not exhibit any immune response to AAV5. A representative figure for one of the peptide pools is presented here **(A–C)**. See Supplementary Figures for details on all pools. Black: control; Magenta: low dose; Cyan: mid dose; Indigo: high dose. *N* = 12 animals per dose group at 1 month, *N* = 8 animals per dose group at 3 months, and *N* ≥ 3 at 6 months post-dosing (total 48 animals at the beginning of the study). Error bars represent the standard deviation in the figure. No immune response was observed against any of the hNR2E3 peptide pools at any time point **(D–F)**. A representative figure for one of the peptide pools is presented here. Each dot represents data for one animal from the GLP toxicology study. Error bars represent the standard deviation in the figure. See Supplementary Figures for details on all pools.

ELISpot is a sensitive immunoassay that evaluates immune B-cell and T-cell responses, as these cells release cytokines when they encounter an immunogen. The cellular immune response to the cytokine interferon gamma (IFN-γ) was evaluated in peripheral blood mononuclear cells (PBMCs), splenocytes, and lymphocytes when these cells were stimulated with different peptide pools of hNR2E3 protein and the AAV5 capsid. Figure 7 shows stimulation of PBMCs (A), spleen (B), and lymph node (C) with a single peptide pool of AAV5 and hNR2E3, respectively. Responses to other AAV5 and hNR2E3 peptide pools are presented in Supplementary Figures S1–S6.

Stimulation of PBMCs, splenocytes, and lymphocytes with AAV5 or hNR2E3 peptide pools (2 and 5 μg/mL) did not elicit an IFN-γ response. In the spleen, most values were within method variability, except for one animal at 3 months and another at 6 months from the high-dose group, which responded to AAV5 peptide pools 3 and 4, respectively. However, there was no immunogenic response observed in PBMCs or lymphocytes in the dosed animals.

## Discussion

AAV5*-hNR2E3* is planned as a treatment for RP in the clinic. To evaluate the delivery and expression of this product in human eyes, we performed biodistribution studies and tested its potential safety and toxicity in the Göttingen minipig eyes, which have a similar anatomy to the human eye (38). Following subretinal administration, the biodistribution of the drug product was assessed in target and non-target tissues. In addition, we evaluated the timing of transgene expression and the persistence of the drug product and protein in the tissues. We observe that the administration of AAV5*-hNR2E3* did not affect general or ocular health in minipigs. Further, no changes in ocular function were observed, and AAV5*-hNR2E3* did not exhibit any adverse effects in the dosed animals.

A biodistribution study is vital to understand drug product pharmacokinetics and distribution in different tissues, which helps establish the safety and efficacy profile for human clinical trials. Our strategy is to specifically transduce the affected retinal cells with the product. To achieve this, the subretinal route of administration was chosen to deliver the product directly to the target photoreceptor and RPE cells. The AAV5 serotype capsid was selected since it is known to effectively transduce retinal cells (43). To achieve a decent level of gene expression, the expression cassette was designed under a synthetic CAG promoter (44, 45).

We did not observe any vector in the tissues from control animals at any time point, demonstrating that there is no interference with the signal from the endogenous (minipig) NR2E3 gene. AAV5*-hNR2E3* was administered to both male and female minipigs, and the vector was not detected in any reproductive tissue (i.e., ovary, testis, or uterus/cervix). As gene therapy products are commonly thought to pose a risk of heritability through incorporation into germinal cells (46). These results demonstrate the safety of the product. In addition, the product was not detected in the heart, kidney, or thymus at any time, and only low levels of the vector were detected at early time points in the blood, lymph node, liver, cerebellum, and cortex. Vector distribution in brain tissues was transient and mostly restricted to the areas related to visual processing (e.g., LGN, optic tract, optic chiasma, and optic nerve). These results demonstrate that off-target distribution of the vector is minimal.

In dosed animals, the vector was detected in target ocular tissues, particularly in the retina and RPE/choroid, where a loss of retinal cells, which is a key feature of RP disease pathogenesis (47, 48). These results demonstrate that the vector is specifically delivered to the target tissues/cells, where it is available to provide therapeutic benefits. The time course analysis of vector distribution showed slow clearance of vector from retinal tissues, which could be attributed to non-internalized extracellular AAV vectors. After subretinal administration, adeno-associated virus (AAV) vectors can persist extracellularly in the retinal space for an extended period due to the relatively immune-privileged and enclosed nature of the subretinal space, as well as limited fluid exchange and enzymatic degradation in this compartment. Some previous studies have reported AAV persistence in animal models as extrachromosomal elements (49, 50). However, over time, tissue levels of AAV vectors are expected to decrease as uninternalized or extracellular AAV vectors are gradually cleared through diffusion, or phagocytosis by resident immune cells such as microglia or retinal pigment epithelium (RPE), or drainage into systemic circulation. This decline reflects the natural clearance processes acting on vectors that have not transduced the target cells, such as vitreous, systemic, and other non-ocular tissues, and tend to minimize off-target effects. This modest reduction in vector levels will not affect the durability of the therapeutic, as transduction of most of the target cells can occur within 2–4 weeks after subretinal dosing. Once the cells are transduced and the transgene has assembled into an episome in the nucleus, it can persist throughout the cell's life.

Positive tissues for vector DNA were further evaluated for NR2E3 protein expression. While protein was detected in several tissues, only a few had an expression above the level of quantification. Notably, the retina exhibited the maximum protein level at 6 months. This expression is expected to persist, as most of the retinal cells are in the post-mitotic phase, and it is expected that after transduction, there will be no loss or dilution in the number of transgene copies. Normally, it is expected that there would be a lag between AAV-mediated transgene delivery and protein expression due to the process of transduction to translation: 1. the product is transduced into the cells; 2. single stranded transgene DNA (ssDNA) template is used to synthesize complimentary DNA; 3. double-stranded DNA (dsDNA) forms episome (circular DNA); 4. mRNA is transcribed from the dsDNA template; and finally, 5. mRNA is translated into protein (51). The kinetics of this process vary and are dependent on factors such as animal species, administration route, vector type, and tissue type. This process is known to be variable and can take a few weeks to months between dosing and the optimal protein expression of the gene therapy product *in vivo*. In this study, the highest protein expression level was observed at 6 months, compared to the 1- and 3-months' time points. This can be attributed to the stabilization and recovery of retinal cells after subretinal dosing and improvement in the health of transduced cells, leading to enhanced expression. This variation may also occur because of variability in animal-to-animal biological responses; variability in the number of transduced cells and the level of transgene copies across groups (1-month group vs. 3-month group vs. 9-month group), and a small sample size. Nonetheless, this study demonstrated sustained expression of NR2E3 protein in target retinal cells, which is crucial for therapeutic benefits in RP patients. In addition, NR2E3 is a transcription factor; we do not anticipate continuous accumulation of this protein inside cells, as it will be subjected to homeostasis mechanisms. NR2E3 belongs to the nuclear receptor superfamily, and many members of this family (e.g., estrogen receptor, androgen receptor, PPARs) are known to be tightly regulated by the ubiquitin-proteasome system (UPS), where proteasomal degradation pathways maintain homeostasis and prevent aberrant transcriptional activity. In addition, native NR2E3 expression is also controlled through feedback mechanisms.

The immune response to a drug is a big safety concern for patients recruited in clinical trials. Recently, a number of patient deaths have been reported for AAV gene therapy products. These products were administered systemically, exposing the patient to a greater risk of widespread immunogenicity. To reduce these risks, patients are often screened for pre-existing antibodies against the AAV capsid and transgene. Pre-existing antibodies are often used as exclusion criteria for patient recruitment in clinical trials, as they may also reduce drug efficacy (52). To overcome this inhibition of efficacy, empty capsids have been administered prior to AAV gene therapy treatment (53). However, it is established now that empty capsids themselves are immunogenic (54). Therefore, a high percentage (>95%) of full capsid is critical to minimize the safety risks.

In the ocular space, AAVs are excellent delivery vectors and are reported to be only weakly immunogenic. The eye is an immune-privileged organ where AAVs are delivered locally, thus minimizing the risk of immune response. However, AAVs have evolved to co-inhabit with humans; many patients may already have neutralized anti-AAV antibodies, which can limit the effectiveness of a gene therapy drug product (55, 56). Based on their serotypes, AAVs have been shown to elicit varying degrees of immune responses. The use of such AAV vectors can induce an immune reaction, raising safety concerns in patients (54, 57). Notably, AAV5 may present a unique case as pre-existing anti-AAV5 antibodies did not appear to reduce drug efficacy. For example, transgene expression of human factor IX (hFIX) was not affected by high anti-AAV5 neutralizing antibodies (NAbs) in seropositive patients (58). Furthermore, an NHP study in which clinical doses were administered to animals seropositive for anti-AAV5 NAbs did not show any impact on transduction efficacy for hFIX protein, supporting the idea that AAV5 has a certain “resistance” to neutralization (59).

Anti-drug antibody (ADA) is a humoral response against a drug, and animals pre-exposed to an antigen mount a greater response that can cause mild to serious immune reactions. Therefore, an ADA response against a drug can be a safety risk and can reduce the drug's efficacy by neutralizing it. For these reasons, determining the ADA response is critical in evaluating the safety and efficacy of clinical trials. In addition, the transgene expression can induce an ADA response, leading to immunogenicity and toxicity (57, 60). In this study, no ADA response was observed against the translated human NR2E3 protein. This is likely due to the high homology (~90%) of the NR2E3 protein between humans and minipigs. Furthermore, no cellular immune response was observed against the AAV5 capsid and hNR2E3 protein in PBMCs, splenocytes, and lymphocytes, indicating that the drug safety and efficacy will not be impacted.

A significant ADA response was observed against the AAV5 capsid in serum and aqueous humor in a dose-dependent manner. Aqueous humor exhibited a somewhat greater response than that in serum. Similar observations have been made for immune response to AAVs in the eye of non-human primates (NHPs); a dose-dependent increase in binding antibodies (BAbs) and neutralizing antibodies (NAbs) was observed in serum post-intraocular dosing in NHPs (61). Importantly, the AAV immune response was not dependent on AAV serotype or route of administration. These results indicate that the eye might not be as immune-privileged as previously believed. In fact, with growing understanding in the field, it appears that the immune-privileged status of the eye and other tissues, such as the CNS, is ambiguous (61–63). Sometimes, the observed higher immune response in the eye could be attributed to its route of administration, as subretinal dosing is an invasive procedure that might trigger the migration of immune cells to the eye, which can collectively mount a great immune response locally. In phase I/II clinical trials for Luxturna (RPE65-LCA), ocular inflammation was noted following subretinal dosing of 1 × 10^12^ vg of AAV2-RPE65 (64, 65). Similarly, we observed a local response in minipig aqueous humor in this study. However, due to the small sample size, these observations are only indicative.

Subretinal delivery, the most common approach for AAV-based gene therapies targeting inherited retinal degenerative diseases, such as retinitis pigmentosa and Stargardt disease, is limited by its invasiveness (requiring vitrectomy, risking endophthalmitis or cataracts), iatrogenic retinal detachment (causing potential tears or atrophy), vector reflux, limited coverage, and inconsistent bleb formation due to retinal biomechanics. Strategies to address these issues include intraoperative OCT for real-time bleb monitoring, automated injection systems that reduce reflux, pre-bleb saline injections for precise placement, and the multi-bleb approach (2–4 injections), achieving 70–80% retinal coverage with lower trauma and immunogenicity. While less invasive suprachoroidal or intravitreal routes are explored, the multi-bleb technique, enhanced by iOCT and robotic precision, optimizes safety and efficacy for retinal therapies.

Ocugen has successfully completed a phase I/II clinical trial (NCT05203939) evaluating the safety and preliminary efficacy of AAV5-hNR2E3 (OCU400) gene therapy product. The results demonstrate a favorable safety profile, with no dose-limiting toxicities or serious adverse events related to treatment. Efficacy analyses revealed encouraging trends across multiple functional vision endpoints, including improvements in best-corrected visual acuity (BCVA), multi-luminance mobility testing (MLMT), and low-luminance visual acuity (LLVA) in treated eyes. Notably, these positive signals were observed across a range of dose levels and genetic subtypes of retinitis pigmentosa (RP), supporting a gene-agnostic mechanism of action. These findings suggest the potential for this therapy to benefit a broader population of RP patients, regardless of the underlying genetic mutation. A Phase III clinical trial (NCT06388200) is currently underway to further evaluate the safety and efficacy of this gene therapy in a larger, more diverse population of patients with retinitis pigmentosa (RP). The trial aims to support the gene-agnostic potential of the therapy across multiple RP genotypes.

## Conclusion

We administered three doses [1.25 × 10^11^ Vg/eye (high); 5 × 10^10^ Vg/eye (mid), 1 × 10^10^ Vg/eye (low)] of AAV5*-hNR2E3* to minipigs via bilateral subretinal dosing. All the doses appear to be well-tolerated. In addition, the product was administered subretinally at a dose of 1.25 × 10^11^ Vg/eye, and biodistribution of the vector was evaluated in ocular and non-ocular tissues. We observed that AAV5*-hNR2E3* is specifically delivered to the target ocular tissues and efficiently transduces retinal cells, where it is translated efficiently to make NR2E3 protein. An ADA immune response against the AAV5 capsid was observed in a few animals at the highest dose, but it did not correlate with clinical observations, and no cellular immune response was observed against AAV5. Based on these results, a clinical dose of 5 × 10^10^ Vg dose/eye was selected as the highest dose for a phase I/II clinical study to provide therapeutic benefit for RP patients.

## Data Availability

The raw data supporting the conclusions of this article will be made available by the authors, without undue reservation.
