# Supplementary material for: Preclinical biodistribution and toxicology assessment of an AAV5-based subretinal modifier gene therapy for retinitis pigmentosa
Source: Front Med (Lausanne). 2025 Oct 29;12:1679619. doi: 10.3389/fmed.2025.1679619 (PMC12605118; doi:10.3389/fmed.2025.1679619)
Supplement: Supplementary file 1 [file Data_Sheet_1.pdf]

Supplementary Fig. 1:

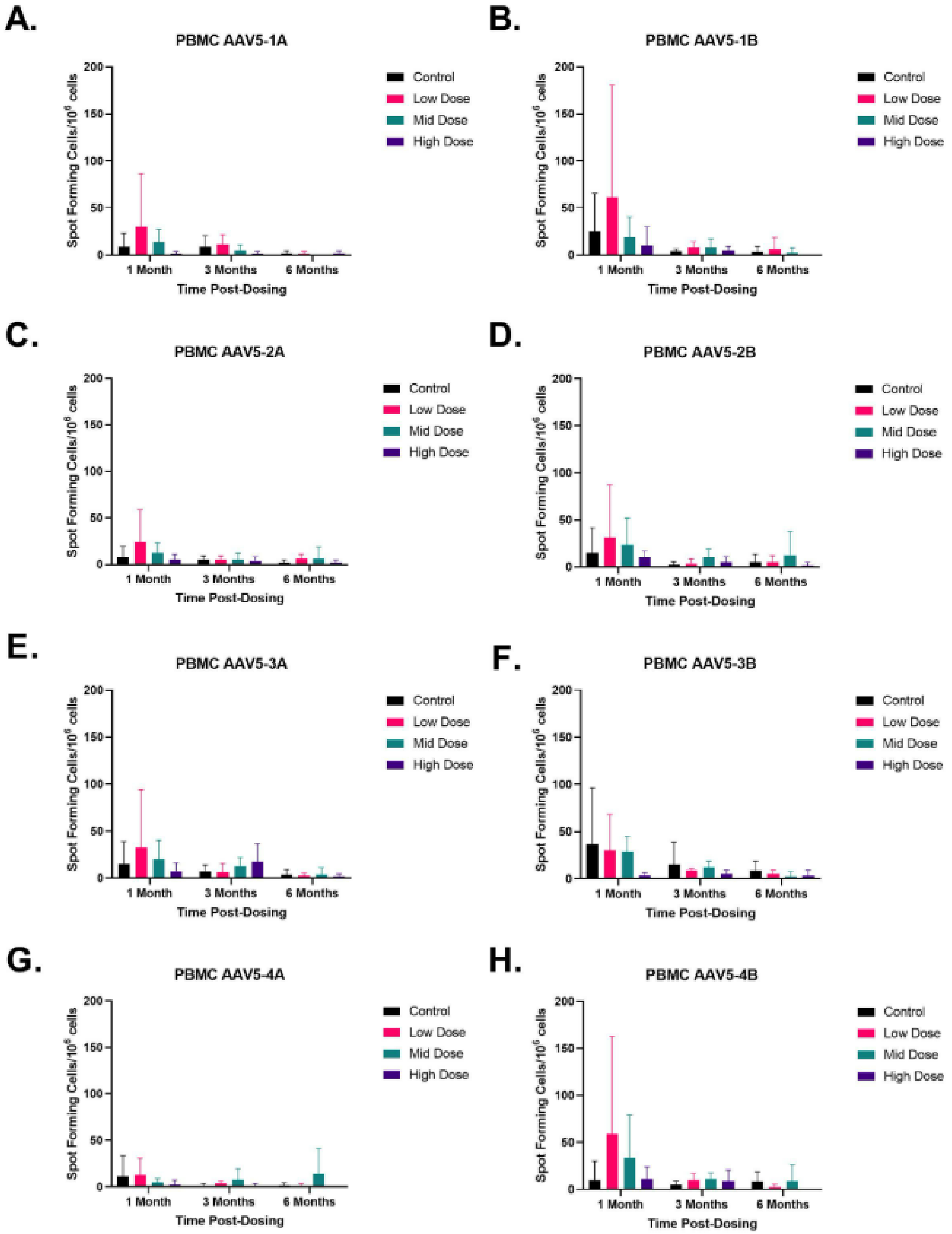

Supplementary Fig. 2:

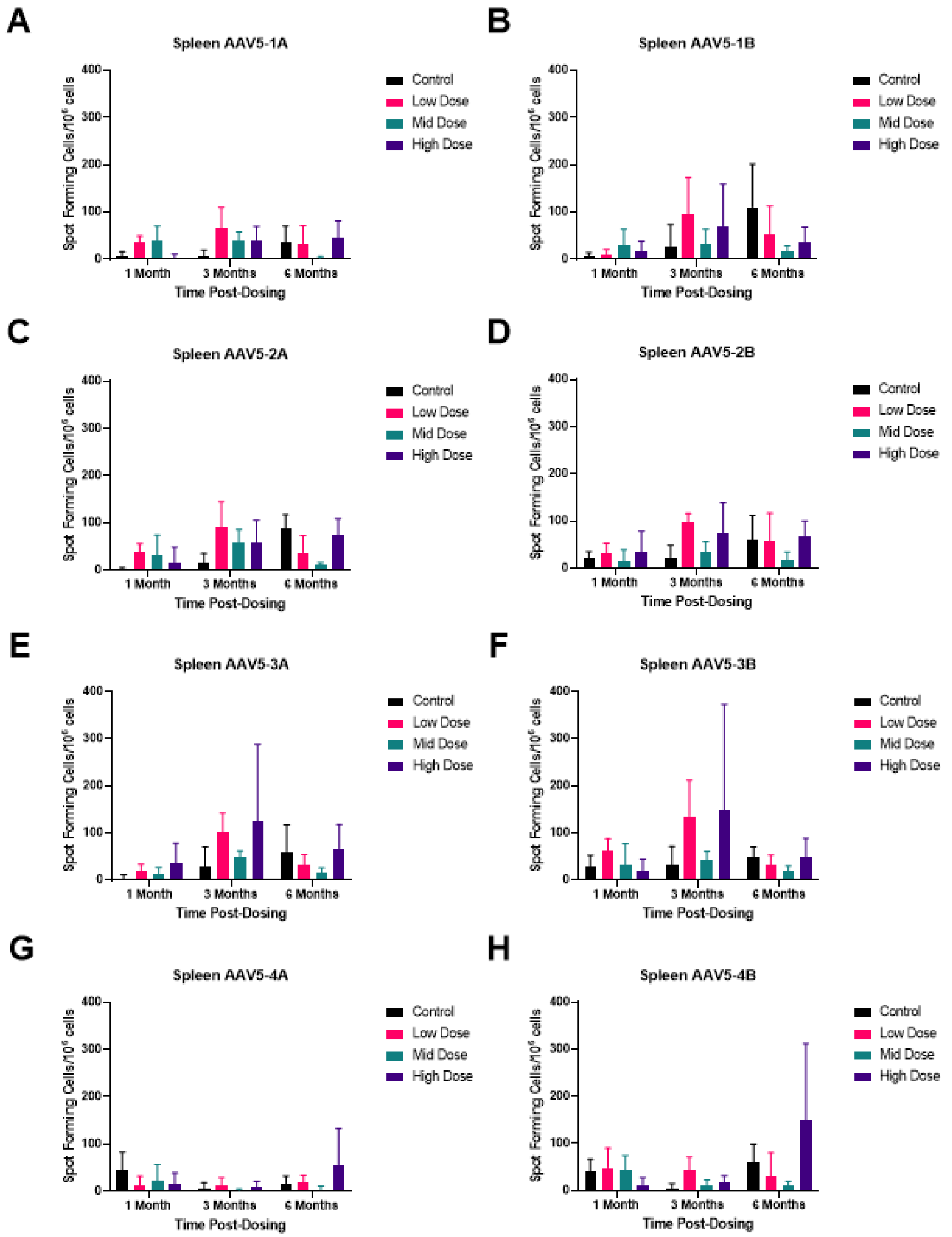

Supplementary Fig. 3:

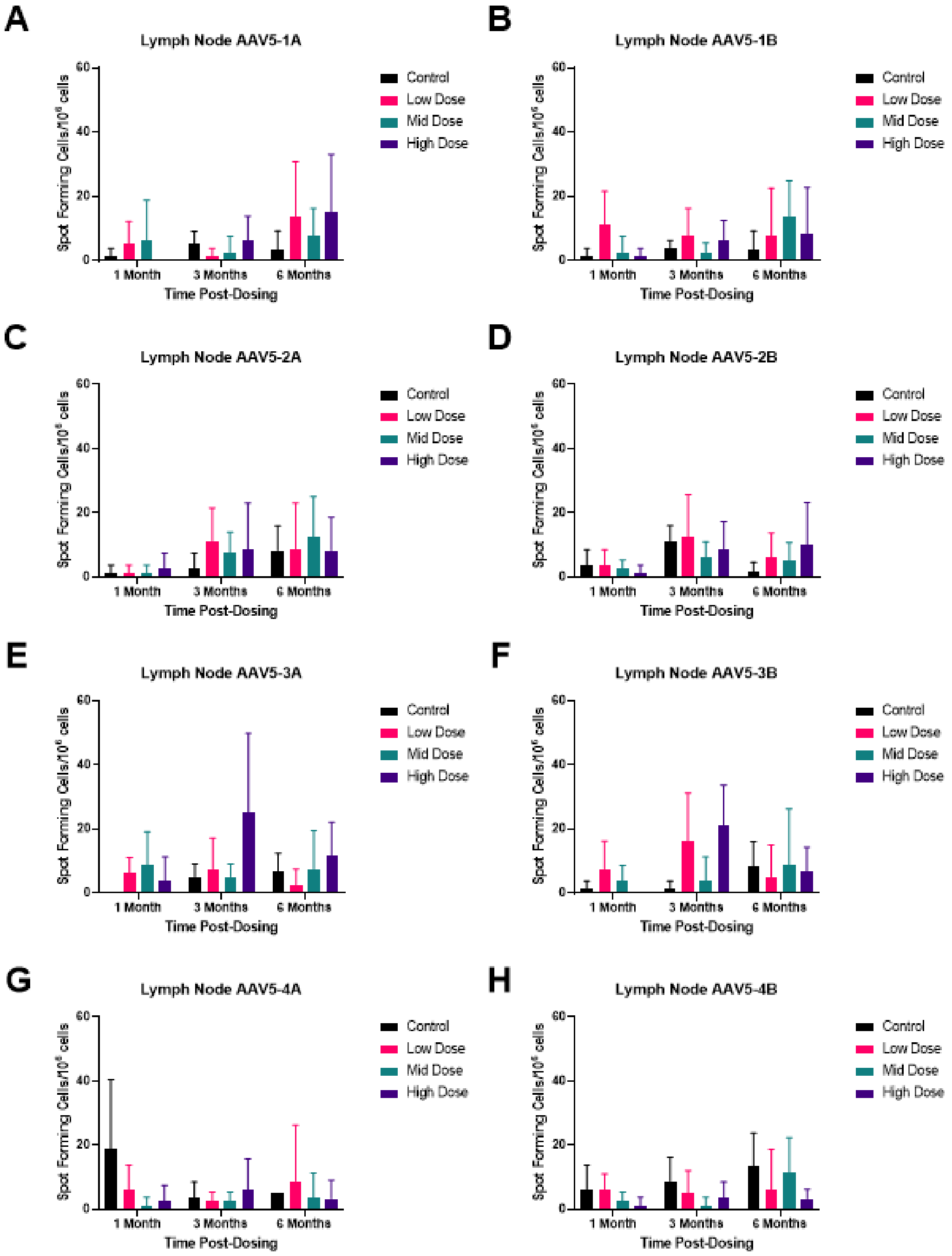

Supplementary Fig. 4:

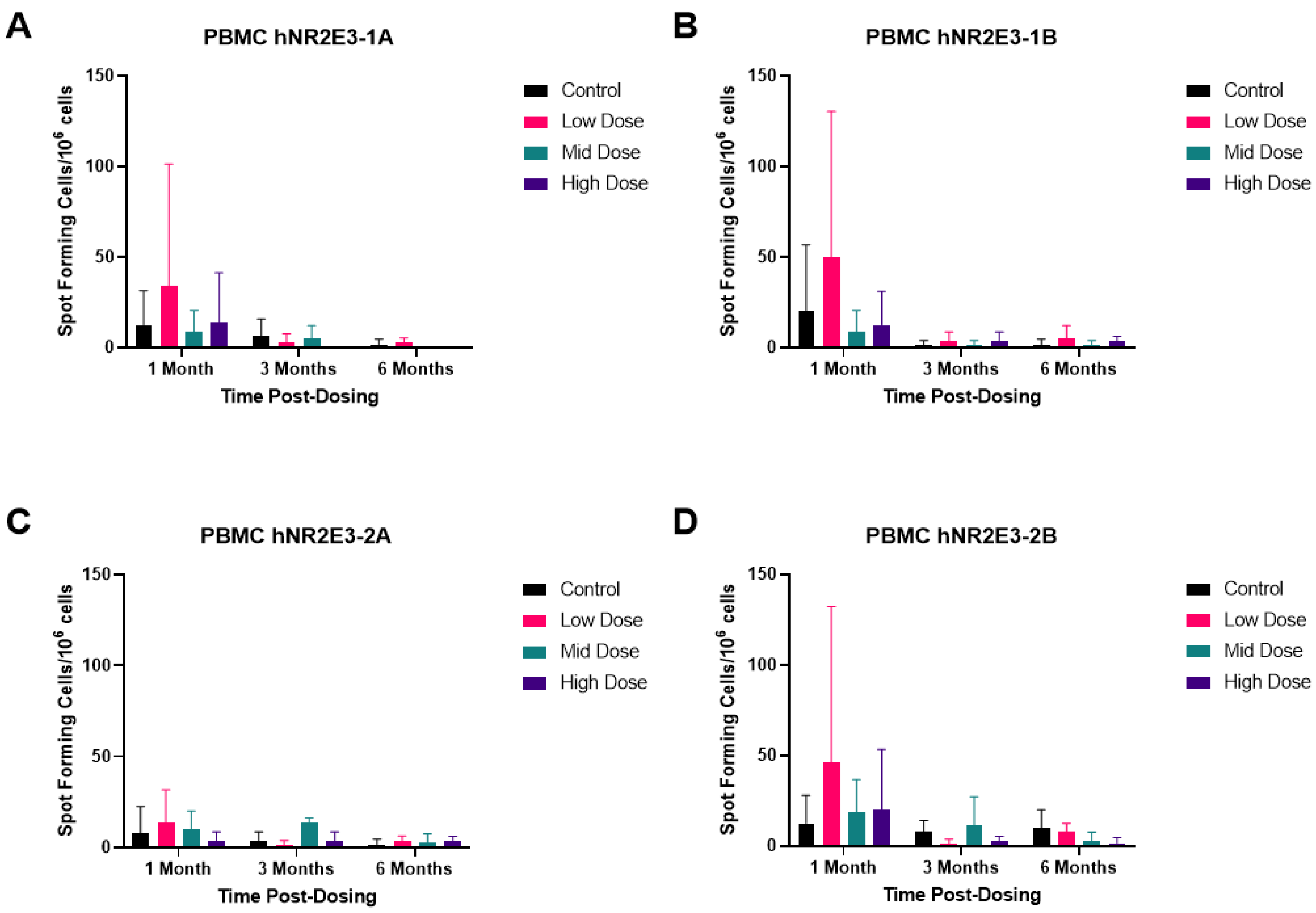

Supplementary Fig. 5:

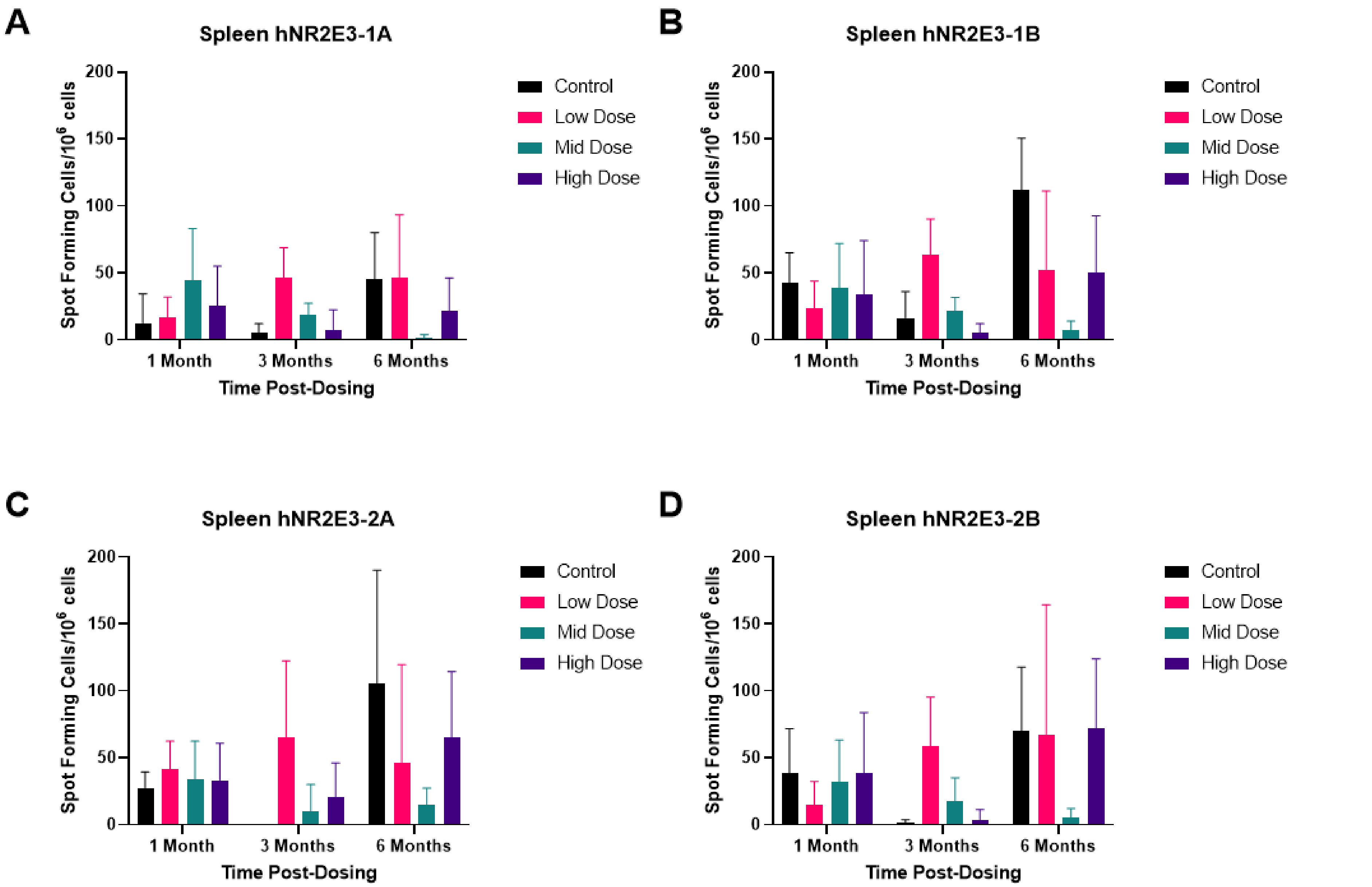

Supplementary Fig. 6:

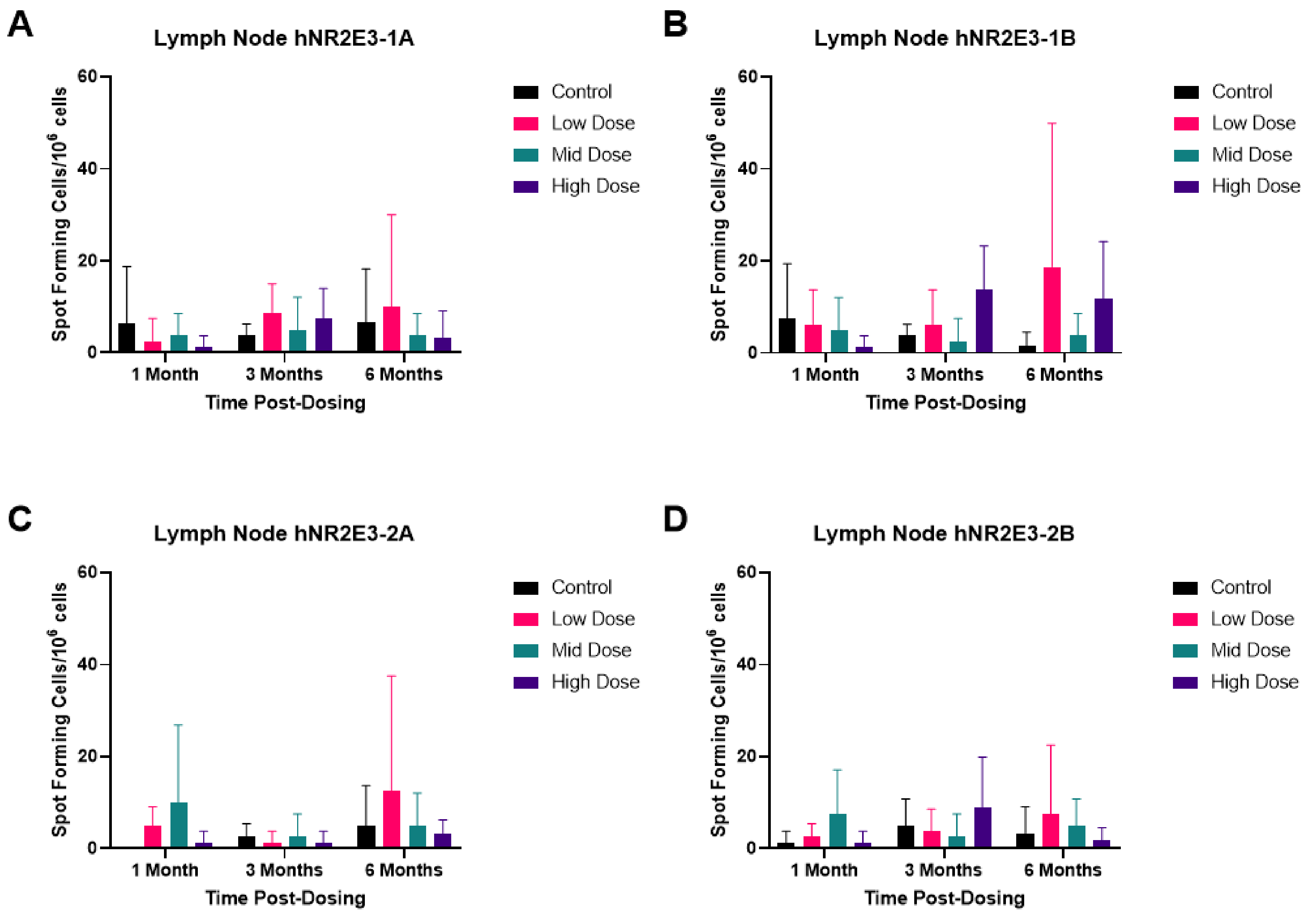

Supplementary figure 1: OCU400 did not elicit an immune response to AAV5 in PBMCs. Eight AAV5 peptide pools were tested for response to IFN-y stimulation. No immune response was detected in any of the pools at 1-, 3-, or 6-months post dosing (A-H). Black: control; Magenta: low dose; Cyan: mid dose; Indigo: high dose.

Supplementary figure 2: OCU400 did not elicit an immune response to AAV5 in splenocytes, except for in 2 animals. Eight AAV5 peptide pools were tested for response to IFN-y stimulation. No immune response was detected in any of the pools at 1-, 3-, or 6-months post dosing (A-H) except for 1 high dose animal at 3 months and 1 high dose animal at 6 months in peptide pool 3 (E and F). Black: control; Magenta: low dose; Cyan: mid dose; Indigo: high dose.

Supplementary figure 3: OCU400 did not elicit an immune response to AAV5 in lymphocytes. Eight AAV5 peptide pools were tested for response to IFN-y stimulation. No immune response was detected in any of the pools at 1-, 3-, or 6-months post dosing (A-H). Black: control; Magenta: low dose; Cyan: mid dose; Indigo: high dose.

Supplementary figure 4: OCU400 did not elicit an immune response to NR2E3 in PBMCs. Eight AAV5 peptide pools were tested for response to IFN-y stimulation. No immune response was detected in any of the pools at 1-, 3-, or 6-months post dosing (A-H). Black: control; Magenta: low dose; Cyan: mid dose; Indigo: high dose.

Supplementary figure 5: OCU400 did not elicit an immune response to NR2E3 in splenocytes. Eight AAV5 peptide pools were tested for response to IFN-y stimulation. No immune response was detected in any of the pools at 1-, 3-, or 6-months post dosing (A-H). Black: control; Magenta: low dose; Cyan: mid dose; Indigo: high dose.

Supplementary figure 6: OCU400 did not elicit an immune response to NR2E3 in lymphocytes. Eight AAV5 peptide pools were tested for response to IFN-y stimulation. No immune response was detected in any of the pools at 1-, 3-, or 6-months post dosing (A-H). Black: control; Magenta: low dose; Cyan: mid dose; Indigo: high dose.

Supplementary Table 1: Incidence of Selected Noteworthy Retinal Changes

	Male and Female			
**Group**	**1**	**2**	**3**	**4**
**Dose (vg/eye)**	**0**	**1.0E+10**	**5.0E+10**	**1.25E+11**
**Ocular Findings**				
Retina Haze/Grayish/Focal/Multifocal/Diffuse	(2)	(1)	(3)	(7)
**No. of Eyes Examined**	**24**	**24**	**24**	**24**
Day 3				
Total Affected	2	1	3	5
Very Slight	2	1	2	3
Slight	-	-	1	2
Day 8				
Total Affected	1	1	2	2
Very Slight	1	1	1	-
Slight	-	-	1	2
Day 15				
Total Affected	0	1	2	2
Very Slight	-	1	2	1
Slight	-	-	-	1
Day 28				
Total Affected	0	0	2	3
Very Slight	-	-	2	2
Slight	-	-	-	1
**No. of Eyes Examined**	**16**	**16**	**16**	**16**
Day 91				
Total Affected	0	0	1	1
Very Slight	-	-	1	1
**No. of Eyes Examined**	**6**	**8**	**8**	**6**
Day 182				
Total Affected	0	0	1	1
Very Slight	-	-	1	1
Retina Elevation/Focal/Multifocal	(2)	(0)	(0)	(0)
**No. of Eyes Examined**	**24**	**24**	**24**	**24**
Day 3				
Total Affected	2	0	0	0
Very Slight	1	-	-	-
Slight	1	-	-	-
Day 8				
Total Affected	1	0	0	0
Slight	1	-	-	-
Day 15				
Total Affected	1	0	0	0
Very Slight	1	-	-	-
Retina/Choroid, Pigment variation from dosing	(20)	(24)	(23)	(23)
**No. of Eyes Examined**	**24**	**24**	**24**	**24**
Day 3				
Total Affected	19	20	20	15
Very Slight	14	15	14	12
Slight	5	5	6	3
Day 8				

Total Affected	18	17	19	18
Very Slight	12	10	8	14
Slight	6	6	7	1
Moderate	-	1	4	3
Day 15				
Total Affected	16	19	22	22
Very Slight	9	11	10	16
Slight	7	6	6	3
Moderate	-	2	6	3
Day 28				
Total Affected	16	24	23	23
Very Slight	7	16	8	8
Slight	9	5	10	12
Moderate	-	3	5	3
**No. of Eyes Examined**	**16**	**16**	**16**	**16**
Day 91				
Total Affected	12	14	14	16
Very Slight	3	8	4	5
Slight	9	5	8	10
Moderate	-	1	2	1
**No. of Eyes Examined**	**6**	**8**	**8**	**6**
Day 182				
Total Affected	6	8	6	6
Very Slight	-	4	1	2
Slight	6	4	5	4
